# Phytochemical Constituents and Pharmacological Potential of *Tamus communis* Rhizomes

**DOI:** 10.3390/molecules27061851

**Published:** 2022-03-12

**Authors:** Iva Slavova, Teodora Tomova, Slavena Kusovska, Yoana Chukova, Mariana Argirova

**Affiliations:** Department of Chemical Sciences, Faculty of Pharmacy, Medical University of Plovdiv, 15A Vassil Aprilov Str., 4002 Plovdiv, Bulgaria; teodora.tomova@mu-plovdiv.bg (T.T.); 20301045@mu-plovdiv.bg (S.K.); 20301071@mu-plovdiv.bg (Y.C.); mariyana.argirova@mu-plovdiv.bg (M.A.)

**Keywords:** *Tamus communis* rhizome, phenanthrenes, sterols, diosgenin, anti-inflammatory, cytotoxicity, cholinesterase inhibitor, xanthin oxidase inhibitor

## Abstract

*Tamus communis* L. is a plant distributed in a number of geographical areas whose rhizome has been used for centuries as an anti-inflammatory and analgesic remedy. This review aims to summarize the current knowledge of the chemical composition and biological activity of the extracts or individual compounds of the rhizome. The data for the principal secondary metabolites are systematized: sterols, steroidal saponins, phenanthrenes, dihydrophenanthrenes, etc. Results of biological tests for anti-inflammatory action, cytotoxicity, anticholinesterase effect, and xanthine oxidase inhibition are presented. Some open questions about the therapeutic properties of the plant are also addressed.

## 1. Introduction

**Figure 1:**
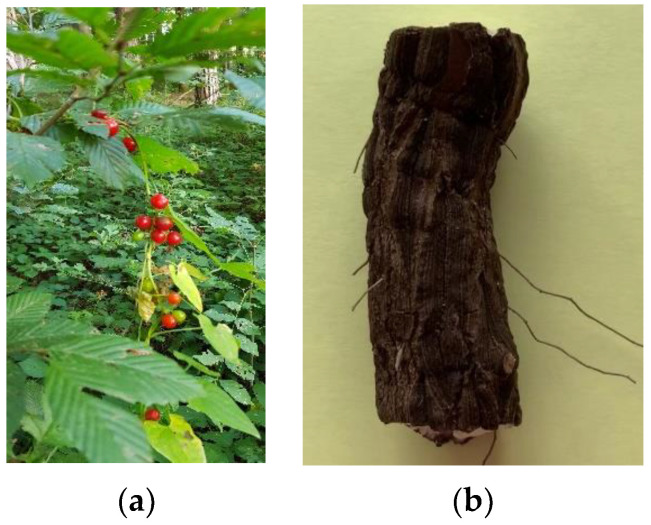
*Tamus communis* L. (**a**) aerial part; (**b**) rhizome (authors’ photos).

*Tamus communis* L., also known as *Dioscorea communis* (L.) Caddick and Wilkin, or black bryony, is a perennial climbing dioecious herbaceous plant with a rhizome, reaching a length of 20–30 cm and a diameter of 5–10 cm (Figure 1). The white, soft core of the rhizome is covered with a thick brown cork layer. It is a very common plant in woods and hedges found almost all over Europe, North Africa, and the Eastern Mediterranean. In Bulgaria—mainly southern Bulgaria—it grows in bushes and young light forests of up to 1200 m altitude [1]. The plant is not protected by Bulgarian legislation.

In Bulgarian traditional medicine, the juice or macerate of the *Tamus communis* L. rhizome (TCR) is applied externally for traumas, rheumatic and muscle pain, sciatica, dioecious herbaceous, and alopecia, and has a beneficial effect on the rapid spread of subcutaneous bleeding. The TCR extract has an irritating effect on the skin, thus improving the blood supply to the affected area [1]. Turkish traditional medicine applies pounded TCR to treat rheumatism [2]. Iraqi folk medicine uses TCR tincture for curing unbroken chilblains [3].

The World Health Organization (WHO) defines traditional medicine as “the total sum of the knowledge, skill, and practices based on the theories, beliefs, and experiences indigenous to different cultures, whether explicable or not, used in the maintenance of health as well as in the prevention, diagnosis, improvement or treatment of physical and mental illness” [4]. The use of plants in traditional and contemporary medicine is most often based on the presence of plant secondary metabolites, compounds that different species synthesize to protect themselves from environmental stress. The main categories of constituents considered to be of therapeutic importance are terpenes, phenolic compounds, alkaloids, etc. [5].

Research on the chemical composition and pharmacological potential of TCR has been of scientific interest for more than half a century. Since nowadays the practical application of plant sources as medicines or nutraceuticals requires detailed information about the chemical composition, the few research groups working on TCR have focused their efforts on elucidating the structure of the main TCR constituents, applying modern instrumental methods such as mass spectrometry and nuclear magnetic resonance, in addition to classical spectral methods. At the same time, the claims of folk medicine about the healing properties of TCR extracts remain largely unsupported by biological tests, and where there are still attempts to prove or deny the healing effect, it is mainly through in vitro experiments. Therefore, despite its wide geographical distribution and centuries-old use in folk medicine, TCR seems to be underestimated as a therapeutic agent plant source and many questions about its pharmacological perspectives remain to be answered.

This minireview aims to summarize the current knowledge about the major classes of secondary metabolites synthesized in TCR, the isolated and structurally elucidated individual compounds, and their biological activity. It is based on an extensive search of the databases Web of Science, Scopus, and Google Scholar using “Tamus communis rhizome/root” as keywords but some information was found in comparative studies with aerial parts (leaves and berries) of the plant. Some data from our research group are also added.

## 2. Chemical Constituents of TCR Extracts

Dried rhizome (*Rhizoma Tami*) is used for medicinal purposes. Plant material is collected after the seeds ripen (September–October) or in early spring during flowering (March–April). In most cases, fresh rhizomes are cleaned, washed, sliced, and air-dried in shade or lyophilized [2,6,7].

Traditional Bulgarian medicine uses TCR extract in olive oil or 70% EtOH (200 g of grated roots in 1 L). The extracts are ready for external use after a 20-day maceration at room temperature in the dark. Aqueous extracts have no therapeutic application.

Moisture in the rhizome accounts for about 3/4 of its fresh weight, which makes it difficult to use hydrophobic organic solvents for the extraction of raw plant materials, but MeOH can be used as an extractant [8]. Direct extraction with non-polar solvents is rarely applied unless the target compounds belong to lipids. Boudjada et al. [9] used diethyl ether for isolation of some bioactive constituents, namely phenanthrenes and dihydrophenanthrenes, from fresh TCR, but the extraction yield is very low, at 0.16%.

MeOH or aqueous MeOH are the preferred solvents for the extraction of polar or moderately polar plant secondary metabolites, mainly phenolic acids and flavonoids, which are very often associated with the biological activity of plant extracts. Kupeli et al. prepared ethanolic and aqueous extracts of TCR using dry plant/solvent ratio 1:10 (two times extraction). The final yield of extractable substances in the organic solvent was 7.6% (*w*/*w*) and aqueous extract yielded 19.8% (*w*/*w*) [2]. This significant difference is understandable because aqueous extracts contain a significant amount of carbohydrates [10], organic acids, and soluble salts. Réthy et al. [11] apply the percolation of fresh rhizomes with MeOH but because of the very high moisture content, the real extractant is aqueous MeOH.

Methods for extracting bioactive components from the rhizome are most often long-term maceration at room temperature with the selected solvent [9,12] or using the Soxhlet apparatus [3,13]. Maceration time also varies, ranging at 2 days [9], 5 days [12], or one week [14].

Information on the extraction yield is not always provided; the few available data show very significant variations in the extractable dry matter depending on the extraction method and the solvent used. The differences in the place and the season of harvesting of TCR probably also contribute to these variations. Mascolo et al. reported 2.8% yield after Soxhlet extraction of powdered dry rhizomes in 80% EtOH [15], Réthy et al. obtained 0.17% dry matter in the extract (fresh plant, CHCl_3_ fraction) [11], and 6.9% extraction yield (dry plant, in MeOH) has also been reported [16].

Usually, after initial extraction with aqueous MeOH, the crude extract obtained is further partitioned with hexane or petroleum ether to remove lipid fraction and then fractioned by successive extraction with CHCl_3_ [11], AcOEt [7], or by solvent–solvent partitioning with petroleum ether, CHCl_3_ and H_2_O [8].

Isolation of individual compounds is typically achieved by silica gel column chromatography using mixtures of CHCl_3_-MeOH with increasing polarity of the eluent [16], gradient system of cyclohexane-AcOEt-EtOH [11], or cyclohexane-AcOEt [9]. Aquino et al. used Amberlite XAD-2 resin and Sephadex LH-20 for the purification of furostanol derivatives from TCR [17].

### 2.1. Phenanthrenes and 9,10-Dihydrophenanthrenes

This type of plant metabolites occurs in numerous plant families, including *Dioscoreaceae,* and have a variety of biological activities [18]. Several research teams have isolated, separated, and elucidated the structure of many polysubstituted methoxy-/hydroxy-phenanthrenes and dihydrophenanthrenes.

The first report about structures of the phenanthrenes isolated from TCR was published in 1969 [19]. The group isolated five phenanthrenes and determined the structure of three of them (compounds **1**, **2**, and **3**, Table 1). Later, the structure of the other two compounds (**4**, **5**) was also elucidated [20]. Some of the proposed structures were revised (**6**, **7**) [21,22]. Most of these compounds had been isolated earlier from other plant species, which has facilitated their identification.

To date, more than fifteen phenanthrenes have been isolated from TCR, which differ in the degree of substitution of the phenanthrene ring, the number of hydroxyl groups, and their methylation (Table 1). Since the synthesis of secondary metabolites is a mechanism that allows plants to adapt to the environment in which they grow, this variety of phenanthrenes largely reflects the influence of local geoclimatic and seasonal characteristics, temperature, humidity, and stage of plant development.

The presence of substituted 9,10-dihydrophenanthrenes in TCR was first reported in 1985 [23]. The compounds **17** and **18** were isolated by preparative TLC of TCR extract in CHCl_3_ and then acetylated to determine the exact position of hydroxyl groups.

Two other dihydrophenanthrenes, compounds **19** and **20**, were isolated for the first time from TCR by Boudjada et al. [9]. Their structures were elucidated using UV, IR, 1D-, 2D-NMR, and MS methods.

### 2.2. Sterols

Extraction of TCR with acetone and further saponification of dried extract with ethanolic KOH yielded sterol fraction [24]. The authors did not give information on the quantity of the sterols; the latter were acetylated and the steryl acetates obtained were separated on silica gel. Retention times, ^1^H-NMR, and MS data were provided for the identified compounds. Chemical analyses showed the presence of β-sitosterol **21** (48.81% of the recovered sterols), stigmasterol **22** (25.12%), and campesterol **23** (23.43%).

### 2.3. Steroidal Saponins

Undoubtedly one of the most intriguing compounds found in TCR is dioscin, whose aglycone is diosgenin (**24**). Both compounds have rich pharmacology, including anti-inflammatory effects [25,26]. In addition, diosgenin is a raw material for testosterone and some steroidal drug syntheses [27].

Dioscin and another known spirostan trioside gracillin were isolated from TCR after chromatographic separation of an extract obtained in CHCl_3_-MeOH [28]. The two saponins represented 0.64% of the extract dry matter and were identified by spectral (^1^H- and ^13^C-NMR, MS) and chromatographic methods (methanolysis and gas chromatographic analysis of silanized methyl glycosides). The exact position of the bonds of carbohydrate residues in gracillin was established by means of partial acid hydrolysis, followed by the remethylation and methanolysis of the glycosides obtained and a comparison of their retention times with that of commercial standards.

The same research group identified three furostanol steroidal saponins isolated from TCR: methylprotoneodiscin (aglycone yamogenin), methylprotodioscin (aglycone diosgenin), and methylprotogracillin. ^1^H-NMR and ^13^C-NMR data were used to identify the compounds [17].

The presence of diosgenin in rhizome was confirmed by HPLC analysis of hydrolyzed TCR extract in 80% EtOH, by comparing the retention time with that of a commercial standard [3]. Other authors performed dioscin hydrolysis before its extraction from the plant material. Hydrolysis with H_2_SO_4_ in isopropanol was the method giving the highest yield of diosgenin. The results obtained showed a higher concentration of diosgenin in the rhizome compared to other plant parts [29].

### 2.4. Phenolic Compounds

Phenolic compounds are the most common type of secondary metabolites in the plant world. Simple phenols, phenolic acids, flavonoids, tannins, and others can be attributed to this group. The frequently reported antioxidant properties of plant extracts are largely associated with the presence of phenolic and polyphenolic compounds [30].

Analyses of different TCR extracts showed the presence of phenolic compounds, including flavonoids. The total content of phenols in the extracts is typically expressed in mg gallic acid equivalents (GAE) in mg per 1 g of dry matter of the extract and varies depending on the extracts used. Reported values for crude methanolic extract are between 26.55 GAE/g [31] and 55.2 GAE/g [14]. Partitioned extracts in CHCl_3_ and AcOEt possess higher content of phenol compounds than crude extract: 78.2 and 61.4 GAE/g, respectively [7].

Flavonoid content expressed in quercetin equivalents (QE) in mg per 1 g dry matter of the extract also varies between crude and partitioned extracts: 8 QE/g for MeOH extract [32] and 10.2 QE/g in AcOEt extract [7]. The content of tannins, another class of phenolic compounds expressed as equivalents of tannic acid (TAE) in mg per 1 g of dry matter [33], has not been reported.

Interestingly, although the phenolic and flavonoid content of extracts was often determined by spectrophotometric methods, the presence of individual phenolic compounds other than those derived from phenanthrene and described in Table 1 has never been reported. We subjected 280 and 360 nm TCR extract in CHCl_3_ to chromatographic analysis with a dual-wavelength absorbance detection using the method developed by Sherova et al. [34]. The quantities of individual aglycones and phenolic acids in the extract are shown in Table 2.

As with many other extracts, the level of flavonoids found by HPLC analysis was significantly lower than the level determined by the spectrophotometric method, which is not surprising since flavonoids in plants are most often glycosylated.

### 2.5. Carbohydrates

We quantified free monosaccharides in methanolic, ethanolic, and mixed (alcohol/water) TCR extracts by HPLC coupled with a refraction index detector (HPLC-RD) using the method developed by Hadjikinova et al. [35]. The most soluble in alcohols monosaccharide, fructose represented 2.4% of the dry matter of extract obtained in 70% MeOH, and glucose and sucrose were 0.7% and 2.2%, respectively. We did not detect any free pentoses in these extracts. Mucoprotein was isolated from TCR, in the composition of which the presence of arabinose, glucose, mannose, and traces of rhamnose was demonstrated, most likely bound to the protein chain [36,37].

### 2.6. Other Constituents

The presence of histamine and acicular crystals of calcium oxalate known as raphides in the TCR was proven by chemical, physical, and pharmacological methods [6,38]. Two furanocoumarins **25** and **26** (5,8-dimethoxypsoralen and heraclinin), were identified by ^1^H-NMR, ^13^C-NMR, and mass spectrometry (MS) [16], but their quantities in the rhizomes were not indicated. The protein content in TCR was low 0.15–0.29% [31].

In a previous study, we reported the concentrations of some essential metal ions (iron, copper, and zinc) in the rhizome [39]. Although these concentrations depend on number of factors (soil composition, environmental pollution, climatic conditions, etc.), an important element is the ability of plant species to synthesize suitable organic ligands to extract these ions from the soil. TCR appears to synthesize specific ligands for zinc ion binding, as the zinc concentration (76.5 ppm) is several times higher than that of iron (20.3 ppm) and copper ions (5.2 ppm).

## 3. Biological Activities of TCR Constituents

### 3.1. Anti-Inflammatory and Analgesic Activity

The most extensive are the studies on anti-inflammatory properties of rhizome extracts, which try to provide scientific evidence in support of centuries of experience in folk medicine. Phytosterols have been shown to have anti-inflammatory properties in both humans and animal models [40]. Because of their non-polar structure, these compounds are well-soluble in non-polar solvents such as CHCl_3_ and hexane, but also in alcohols, acetone, and AcOEt [41]. It can be assumed that olive oil used in traditional medicine extracts a significant part of the sterols in the rhizome, but other solvents used in experimental studies may also have phytosterols as principal bioactive constituents.

TCR extract in 80% ethanol was tested for anti-inflammatory and analgesic properties [13]. The dried extract was dissolved in gum arabic (5%) as a vehicle and administrated orally to rats. In a model of acute carrageenan-induced inflammation, the extract showed a dose-dependent reduction in the foot paw swelling of rats that was commensurate to the anti-edematous effect of anti-inflammatory drug phenylbutazone. In the model of cotton pellet-induced granuloma, the extract was as potent as phenylbutazone. The analgesic effect of TCR extract has been studied and demonstrated in a model of acute pain induced by the intraperitoneal injection of acetic acid in mice. TCR extract administered orally at doses of 60 and 120 mg/kg resulted in a significant reduction in seizure episodes compared to controls. The results were very close to those obtained with the positive control phenylbutazone. In this study, LD_50_ 800 mg/kg was found, which means that the extract has a moderate therapeutic index. The authors attribute these anti-inflammatory and analgesic properties to the presence of campesterol, stigmasterol, and β-sitosterol in the extract.

This hypothesis was further tested in a model of carrageenan-induced edema in rats applying extracts of different parts of the plant. It was observed that the effect of edema reduction was less pronounced applying fruit extract compared to rhizome and leaf extracts. On the other hand, larger quantities of sterols were found in the TCR than in the fruits. Based on this, it was assumed that sterols are involved in the anti-inflammatory effect of TCR extract [24].

Using cotton pellet-induced granuloma as a model of subacute inflammation, 80% ethanolic extract was impregnated in the implanted cotton pellets (5–20 mg/pellet) [15]. Suppression of granule formation by TCR extract was compared with the anti-inflammatory drugs benzydamine HCl (5–20 mg/pellet) and hydrocortisone (5 mg/pellet). While a dose of 1 mg/pellet did not inhibit the formation of granulomas, doses of 5, 10, and 20 mg/pellet inhibited granuloma formation by 12, 27, and 49%, respectively. Benzydamine, at doses of 5–20 mg/pellet, resulted in identical to the extract suppression of granuloma formation (16–50% inhibition), while hydrocortisone at doses of 5 mg/pellet showed 64% efficacy. The authors proposed that the TCR extract prevents the synthesis or release of prostaglandins, the well-known mediators of inflammation, by rat peritoneal leucocytes.

The local anti-inflammatory effect of TCR was also studied by the transdermal application of 1% and 2% (*w*/*v*) EtOH extract to rat foot paw in a model of carrageenan-induced acute inflammation. Sodium diclofenac (1.5% alcohol solution) with a proven anti-inflammatory effect was used as a positive control. The results obtained showed a dose-dependent effect: after 24 h, 1% TCR extract demonstrated a comparable anti-inflammatory effect to that of diclofenac, and 2% extract showed a more pronounced local anti-inflammatory effect than the used nonsteroidal anti-inflammatory drug [42]. This study, however, showed that the TCR extract was less effective on chronic inflammation (cotton pellet-induced granuloma) and did not prove any analgesic effect.

Phytosterols are practically insoluble in water, and this could explain the findings in another study that compares the anti-inflammatory and analgesic (antinociceptive) effects of ethanolic and aqueous extracts prepared from eleven plants used in Turkish folk medicine [2]. Both extracts were applied orally in doses of 250 and 500 mg/kg suspended in aqueous 0.5% sodium carboxymethyl cellulose. According to this study, the TCR extracts demonstrated weaker anti-inflammatory and analgesic activity compared to the other plants studied. Ethanolic extract was more potent than aqueous one, the highest applied dose: 500 mg/kg reduced 10.5% of carrageenan-induced rat paw edema and inhibited abdominal contractions caused by p-benzoquinone by 18.4%.

Amraoui et al. evaluate the anti-inflammatory and anti-arthritic effects of TCR methanolic extract [14]. Since the three-day oral administration of the dried extract (100 mg/kg) did not show any toxic symptoms or mortality, higher doses of 150, 300, and 600 mg/kg were applied orally, but these did not demonstrate anti-inflammatory and anti-arthritic effects in animal models compared to the not-treated control.

Overall, these data show that pharmacological studies conducted to confirm or rule out the healing properties of the TCR claimed by traditional medicine are contradictory and incomplete. Perhaps a number of factors influencing the possible anti-inflammatory activity remain to be clarified: the most appropriate extractant, the most appropriate method of administration, and the probable mechanism of action, if any.

### 3.2. Cytotoxicity

Biosynthesis of both phenanthrenes and dihydrophenanthrenes starts from aromatic amino acids, but the immediate precursor of phenanthrenes is stilbene, and bibenzyl is the predecessor of dihydrophenatrenes [43]. Synthetic and natural stilbene derivatives have been reported as efficient anticancer agents [44]. Since phenanthrenes can be considered as cyclized cis-stilbenes, the interest in their potential cytotoxicity is reasonable.

Four phenanthrenes and one dihydrophenantrene isolated from TCR petroleum ether extract were tested in the cervix adenocarcinoma (HeLa) cell line and their cytotoxic effect was compared with the effect of the anti-tumor drugs, cisplatin and doxorubicin, using MTT (3-(4,5-dimethylthiazol-2-yl)-2,5-diphenyltetrazolium bromide) assay [8]. Only one of the tested compounds was not active in suppressing tumor cell growth; the others displayed pronounced cytotoxic activity with compound **14** (Table 1) being the most active in the decrease in cell viability after 3 days of treatment.

Other two cell lines: human epidermoid laryngocarcinoma (HEp-2) and mammary adenocarcinoma (AMN-3) were used to compare the cytotoxicity of TCR extract and pure diosgenin [3]. After extraction with 80% EtOH, and acid hydrolysis, the presence of diosgenin in TCR extracts was proven by HPLC. Plant extract in concentrations 125–500 µg/mL demonstrated a better cytotoxic effect in the HEp-2 cell line than pure diosgenin applied in the same concentrations, but the latter was more cytotoxic to the AMN-3 cell line than the plant extract.

### 3.3. Antibacterial Properties

One of the functions of plant secondary metabolites is defending species against an attack of pathogens. In search for a replacement of synthetic chemical products with natural, many plant extracts and individual plant constituents have been tested for antimicrobial activity [45].

CHCl_3_ and AcOEt extracts obtained after the partitioning of crude aqueous-methanolic TCR extract demonstrated weak antibacterial properties against four (*Staphylococcus aureus*, *Bacillus subtilis*, *Enterobacter agglomerans*, and *Serratia marcescens*) of a total of nine strains tested but this activity was weaker than the activities of pure phenolic compounds: rutin, quercetin, and gallic acid (their concentrations are not shown) [7]. The authors considered the presence of phenolic compounds in the extract as a reason for the manifestation of antibacterial properties.

### 3.4. Anti-Viral Effect

Twelve compounds isolated from TCR (four phenanthrenes (**1**, **2**, **3**, and **4**), two dihydrophenanthrenes (**17**, **18**), two spirostane triglycosides (dioscin and gracillin), and four furostane tetraglycosides) were tested for their potential antiviral activity [46]. The inhibitory effect on viral replication was studied on two RNA viruses: vesicular stomatitis virus (VSV) and human rhinovirus serotype 1B (CRO 1B). The results of the screening investigation reported in this study revealed a weak antiviral activity demonstrated mainly by phenanthrene and dihydrophenantrene derivatives; they were more active toward VSV than HRV. The spirostane and furostan derivatives demonstrated an inhibitory effect at doses close to the maximum non-toxic dose of 100 μg/mL and their effects were considered by the authors as “not interesting results” [46].

### 3.5. Xanthin Oxidase Inhibition

Xanthine oxidase (XO) is a molybdenum-containing flavoprotein that catalyzes the oxidation of hypoxanthine and xanthine to uric acid in purine catabolism. In addition, XO-catalyzed reactions produce reactive oxygen species (ROS) that may contribute to tissue damage. Overactivity of XO and/or retarded excretion of uric acid leads to the deposition of monosodium uric acid crystals in kidneys, joints, and other tissues. The condition, known as hyperuricemia, is considered a risk factor for the development of gout, hypertension, type 2 diabetes, chronic kidney disease, and cardiovascular disease. [47,48]. A therapeutic approach to prevent the development of hyperuricemia-related disorders is to use XO inhibitors that block the biosynthesis of uric acid. Nowadays, the identification of xanthine oxidase inhibitors of natural origin is of increasing interest. Potential candidates for such inhibitors are flavonoids and tannins [49,50].

TCR extracts prepared in different solvents (MeOH, CHCl_3_, and AcOEt) were tested for their ability to inhibit the enzyme and scavenge superoxide anion radical (O_2_^•−^) generated by the XO [31]. Data obtained indicated that the most effective was the extract in AcOEt. The authors proposed that some compounds in the extracts act rather as antioxidants scavenging superoxide radical, while other compounds inhibit XO.

Further on, the same research group reported the identification and isolation from TCR of two strong XO inhibitors, which were identified as 5,8-dimethoxypsoralen and heraclinin (compounds **25** and **26** in Table 1) [16]. Both compounds inhibited XO in a concentration-dependent manner, with IC_50_ values of 0.074 ± 0.003 and 0.099 ± 0.002 mg/mL for heraclinin, and 5,8- dimethoxypsoralen, respectively. These compounds were also effective as superoxide radical scavengers, but it was not possible to unambiguously prove whether the reduced levels of the radical in the assay used are due to furanocoumarins inhibiting an effect on XO.

The relationships between the content of phenolic compounds in plant extracts, their antioxidant activity, and inhibition of XO was recently documented [32]. TCR crude extract in 85% MeOH was further separated using column chromatography into six fractions eluted with different ratios CHCl_3_/MeOH. The authors compared total phenolic content, flavonoid content, XO inhibition, and the superoxide radical scavenging ability of the six fractions and, based on the results obtained, concluded that capacity in inhibition of XO and O_2_^•−^ scavenging activity of the studied fractions depends on the levels of phenolic compounds.

### 3.6. Effect on the Activity of Acetylcholine Esterase

Alzheimer’s disease (AD), which accounts for about 75% of all dementia cases, is a progressive neurodegenerative disorder leading to memory loss and various behavioral disorders [51]. The disease is characterized by low levels of the neurotransmitter acetylcholine (ACh) in the brain and, therefore, a possible treatment of AD is associated with increasing the amount of ACh in the cholinergic synapses by inhibiting the two types of cholinesterase enzymes (acetylcholinesterase AChE and butyrylcholinesterase BChE). To date, several synthetic AChE inhibitors (tacrine, donepezil, rivastigmine, galantamine) are known to delay ACh hydrolysis, but clinical evidence shows that these drugs may cause a number of side effects, such as gastrointestinal disturbances, hepatotoxicity, and problems associated with bioavailability. Numerous plant extracts have been subjects of extensive research in finding better AChE inhibitors with potential application for the treatment of various neurological disorders [52,53].

Crude TCR diethyl ether extract and four individual phenanthrenes were tested for their ability to inhibit the cholinesterase enzymes (AChE and BChE) [9]. Only one of the tested phenanthrenes, compound 11, Table 1 (2,4,8-trimethoxy-3,7-phenanthrenediol), inhibited the activity of AChE (IC_50_ = 69.41 µg/mL) and its effect was close to those of the well-known AChE inhibitor galantamine (IC_50_ = 94.77 µg/mL). However, a compound that was identified as 2,4-dimethoxy-7,8-methylendioxy-3-phenanthrenol (**14**), and the crude extract in diethyl ether, exhibited anti-BChE activity (IC_50_ = 11.40 μg/mL and 14.34 μg/mL, respectively) that was three times higher than the activity of galantamine (IC_50_ = 34.75 μg/mL).

### 3.7. Skin Irritation

Skin reactions in ten volunteers 30 min and 24 h after rubbing *Tamus communis* fruit juice and TCR mucilage was compared [6]. The material from the rhizome causes a more intense reaction on contact with the skin, compared to the juice from the fruit. According to the authors, the rhizome mucilage leads to mechanical and chemical irritation associated with calcium oxalate crystals in mucilage and intracutaneous injection of histamine, whereas with fruit juice, the irritation is only mechanical.

### 3.8. Other Activities

Several diseases and pathological conditions are accompanied by the overproduction of ROS, and this leads to oxidative stress in the body. It has been assumed that plant antioxidants taken through food or as dietary supplements may alleviate oxidative stress. However, the methods used to evaluate these antioxidant properties of plant extracts use conditions that are very different from the physiological ones; there is a large variation in presenting the results obtained, which makes it difficult to compare data for different plant species, and the usefulness of these results is still very debatable [54].

TCR extract in 80% MeOH obtained from dry plant material was further partitioned against solvents with different polarity and their in vitro antioxidant activity was measured applying two methods: DPPH (2,2-diphenyl-1-picrylhydrazyl) free radical scavenging capacity and β-carotene bleaching [7]. Non-polar fractions demonstrated significantly higher scavenging activity than polar; the antioxidant properties of CHCl_3_ extract were comparable to that of the positive control butylated hydroxytoluene (BHT). The authors accredit these antioxidant properties to the presence of phenolic compounds in the extracts since five tested individual phenols (rutin, catechine, gallic acid, caffeic acid, and tannic acid) demonstrated comparable to those of BHT inhibition of linoleic acid oxidation.

An in vivo evaluation of the antioxidant properties of TCR extract in mice has been carried out by Zerargui et al. [12]. Crude extract in 85% MeOH was applied intraperitoneally at a dose of 100 mg/kg/day for 21 consecutive days; a second group was treated with vitamin C (50 mg/kg). There was a significant decrease (54.09%) in the content of malondialdehyde in the liver as a lipid peroxidation marker and an increase in glutathione concentration and catalase activity by 47.30% and 46.87%, respectively, in the group treated with TCR extract compared with the negative control group that received only saline solution. Comparison between markers used in this study for the group treated with TCR extract showed better improvement of antioxidant status than that of the positive control group loaded with vitamin C.

The specific biological activities of extracts in different solvents and individual extract constituents are summarized in Table 3.

## 4. Molecular Mechanisms Underlying the Biological Effects of Some *Tamus communis* Constituents

Although the exact molecular mechanisms of the anti-inflammatory, cytotoxic and other pharmacological effects of *Tamus communis* extracts and some pure isolates shown in Table 3 have not been reported, some mechanisms have been clarified for similar compounds, albeit isolated from other plant sources.

### 4.1. Phytosterols

Structural similarity to cholesterol allows the phytosterols delivered through daily diet to competitively reduce the absorption of cholesterol in the gastrointestinal tract to lower low-density lipoproteins levels (LDL), and thus to reduce the risk of cardiovascular diseases [55]. Apart from their hypocholesterolemic activity, plant-derived sterols exert an anti-inflammatory effect that has been proven by numerous experimental and clinical studies [40]. Most often, the anti-inflammatory drugs operate by inhibiting the production of pro-inflammatory enzymes: inducible cyclooxygenase (COX 2) and inducible nitric oxide synthase (iNOS) and pro-inflammatory cytokines (tumor necrosis factor (TNF)-α, interleukin (IL)-1, IL-6). Recently, a study on some phytosterols, including β-sitosterol **21**, stigmasterol **22**, and campesterol **23**, found in TCP extract as well, showed that the first two compounds significantly suppressed the expression and activity of COX and iNOS. These enzymes are associated not only with inflammatory diseases but also with carcinogenesis [56].

### 4.2. Phenanthrenes and Dihydrophenanthrenes

Many of these natural polycyclic aromatic compounds have demonstrated anti-inflammatory potential. An in vitro study showed that 2,7-dihydroxy-4,6-dimethoxy phenanthrene isolated from *Dioscorea batatas* exerted an anti-inflammatory effect by suppressing the expression of COX-2, iNOS, and nuclear factor kappa B (NF-κB)-mediated inflammation [57]. Inhibition of COX enzymes by four individual phenanthrenes (2,6-dihydroxy-4,7-dimethoxyphenanthrene, 6-hydroxy-2,4,7-trimethoxyphenanthrene, 2,7-dihydroxy-4,6-dimethoxyphenanthrene, and 6,7-dihydroxy-2,4-dimethoxyphenanthrene) isolated from *Dioscorea opposita* was confirmed as the principal mechanism of their strong anti-inflammatory activity; higher or commensurable to that of aspirin, ibuprofen, and naproxen [58].

Phenanthrenes and dihydrophenanthrenes, isolated from various plant species, including *Tamus communis*, have shown cytotoxic properties on specific tumor cell lines [43], but this in vitro effect may have different underlying mechanisms: disrupture of cell membranes, impairment in cellular metabolism, DNA damage, or combinations of these. Apoptosis induction in cancer cells is one of the main anticancer strategies and a common mechanism of action of several anticancer drugs, but there are many apoptotic mediators leading to programmed cell death.

Juncunol (7-ethenyl-1,6-dimethyl-9,10-dihydrophenanthren-2-ol) isolated from *Juncus acutus* induced cellular apoptosis in human hepatocellular carcinoma cell line HepG2 [59]. The same mechanism for cytotoxicity was demonstrated by other phenanthrenes: denbinobin (5-hydroxy-3,7-dimethoxyphenanthrene-1,4-dione), fimbriol B (2,3,5-trihydroxy-4-methoxyphenanthrene), and 2,3,5-trihydroxy-4,9-dimethoxyphenanthrene, which showed a strong apoptosis-inducing capacity on hepatic cells [60]. Nam et al. studied the cytotoxic effect of nine phenanthrenes and 9,10-dihydrophenanthrenes on the human hypopharynx squamous carcinoma cell line and, from the results obtained, deducted that methylation of the phenolic groups or their oxidation to oxo-groups increases the cytotoxic activity [61]. In contrast, Lee et al. studied the cytotoxicity of 4,7-dihydroxy-2-methoxy-9,10-dihydrophenanthrene (**20**) and denbinobin, and found that their synthetically methylated and acetylated derivatives did not exhibit in vitro and in vivo antitumor activity [62].

### 4.3. Furanocoumarines

This group of natural heterocyclic compounds attracted researchers’ attention with its ability to act as photosensitizers, i.e., to produce singlet oxygen or superoxide radical upon UVA radiation. These reactive oxygen species interact with cellular membranes, proteins, and DNA, and many therapeutic properties result from their photosensitivity. Phototherapy with furanocoumarins has been considered promising for some skin diseases such as vitiligo, psoriasis, systemic lupus erythematosus, mycosis, fungoides, Sézary syndrome, and pemphigus vulgaris [63]. The furanocoumarin phototoxicity is proposed to result from covalent binding between furanocoumarins and one or two pyrimidine bases under UVA radiation leading to cell cycle arrest and induction of apoptosis.

The non-substituted linear furanocoumarin psoralen is the most studied compound of this group of natural compounds that possesses anticancer activity on number of tumor cells. Depending on the cell line used, cell cycle arrest in G1/S, G1/G0, or G0/G1 phase was proposed as the mechanism of the registered cytotoxicity, suggesting that this effect may be tumor-specific [64]. Derivatives of psoralen, namely 5- and 8-methoxy substituted psoralenes demonstrated enhanced pro-apoptotic and anti-proliferative activity in human leukemia cells compared to the parent psoralene [65].

These studies on the molecular basis of the biological effects of phytosterols, phenanthrenes, dihydrophenanthrenes, and furanocoumarins show that the mechanisms of their action largely coincide or complement each other, and it is very likely in TCR extracts the individual constituents to exert a synergistic effect.

## 5. Closing Remarks

The idea of using natural products as an alternative to steroidal and non-steroidal anti-inflammatory drugs to treat chronic diseases is tempting and deserves more extensive research. Some natural products such as curcumin from the rhizomes of *Curcuma longa* L., epigallocatechin-3-gallate from *Camellia sinensis*, tea catechins, and several plant extracts are in clinical trials in humans with osteoarthritis and rheumatoid arthritis [66]. TCR extracts could be such an alternative, and this largely determines the research interest in this plant. It is widespread in various geographical areas, non-endangered, available, and renewable sources. TCR principal constituents that would have a medical application are well-established and belong to different classes of secondary metabolites: sterols, steroidal saponins, phenanthrenes, dihydrophenanthrenes, phenolic acids, and flavonoids. However, even if these compounds are orally administrated and absorbed in the gastrointestinal tract, their low solubility in water would not allow them to be transported to the target tissues in therapeutic concentrations. Therefore, based on a century of experience of folk medicine, they are more likely to have a healing effect when applied topically than orally.

The available data, although obtained from in vitro experiments or animal models, support the anti-inflammatory effect of the extracts, but the exact molecular mechanism remains unclear. The authors of these studies attribute this effect to the presence of sterols, which are far from unique to this plant species, and the information about their content in TCR is scarce. It can be assumed that the compounds possessing cytotoxic activity (phenanthrenes and saponins) also contribute to the anti-inflammatory action. The data obtained for cytotoxic and anticholinesterase activity of some individual phenanthrenes are a good basis for structure–activity relationship studies.

Furanocoumarins, whose antioxidant, anti-inflammatory, and anti-proliferative properties have been the focus of number of studies, with their prospective pharmacological applications having been recently reviewed [67], also deserve more in-depth research. Their contribution to the anti-inflammatory effect demonstrated by the extracts remains unclear.

Despite the collected data shedding light on the chemical composition and pharmacological potential of the TCR, they are incomplete and fragmented, and the scientific validity of some of the reported biological effects must be proven at least in animal models. Further studies are needed on the mechanism of documented bioactivities, optimization of the methods for extraction, and their standardization in terms of active components, which would allow achieving comparability of the results obtained by different research groups, as well as finding the most effective form of application of the isolated bioactive substances to maximize their therapeutic effect.

## Figures and Tables

**Table 1:** Compounds isolated from organic extracts of *Tamus communis* rhizome.

Compound	Data	Reference
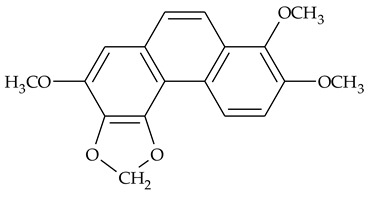 2,7,8-trimethoxy-3,4-methylenedioxyphenanthrene **(1)**	m.p., UV, ^1^H-NMR	[19]
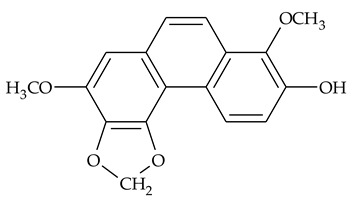 2,8-dimethoxy-7-hydroxy-3,4-methylenedioxyphenanthrene **(2)**	m.p., UV, ^1^H-NMR	[19]
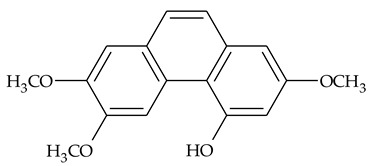 5-hydroxy-2,3,7-trimethoxyphenanthrene **(3)**	m.p., UV, ^1^H-NMR	[19]
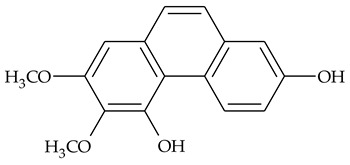 4,7-dihydroxy-2,3-dimethoxyphenanthrene **(4)**	^1^H-NMR	[20]
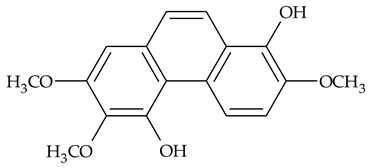 4,8-dihydroxy-2,3,7-trimethoxyphenanthrene **(5)**	^1^H-NMR	[20]
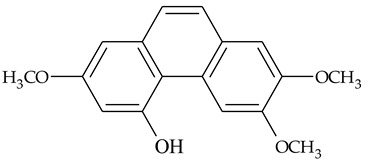 4-hydroxy-2,6,7-trimethoxyphenanthrene **(6)**	m.p., ^1^H-NMR	[21]
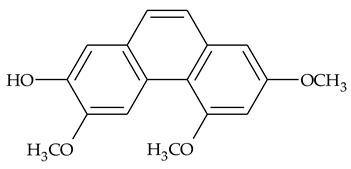 Isobatatasin I **(7)**	m.p., ^1^H-NMR	[21]
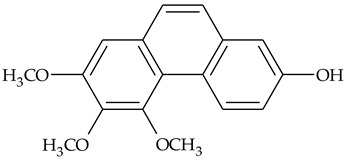 7-hydroxy-2,3,4-trimethoxyphenanthrene **(8)**	m.p., UV, MS, ^1^H-NMR	[11]
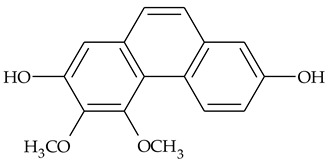 Nudol **(9)**	m.p., UV, MS, ^1^H-NMR	[11]
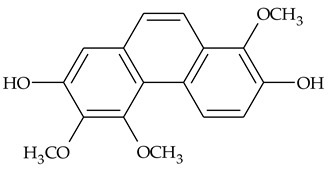 Confusarin **(10)**	m.p., UV, MS, ^1^H-NMR	[11]
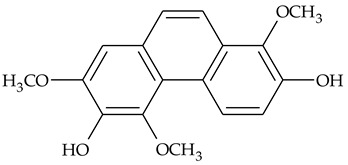 3,7-dihydroxy-2,4,8-trimethoxyphenanthrene **(11)**	m.p., UV, MS, ^1^H-NMR	[11]
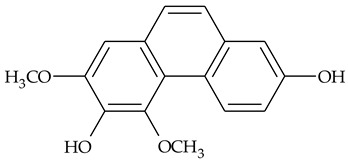 3,7-dihydroxy-2,4-dimethoxyphenanthrene **(12)**	m.p., UV, MS, ^1^H-NMR	[11]
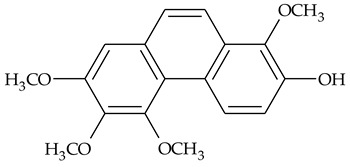 Chrysotoxene **(13)**	UV, ^1^H-NMR, ^13^C-NMR, MS,	[8]
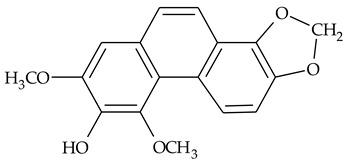 3-hydroxy-2,4-dimethoxy-7,8-methylenedioxyphenanthrene **(14)**	UV, ^1^H-NMR, ^13^C-NMR, MS,	[8]
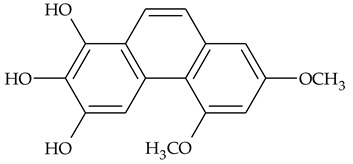 Herorensol **(15)**	R_f_, ^1^H-NMR, ^13^C-NMR, MS	[9]
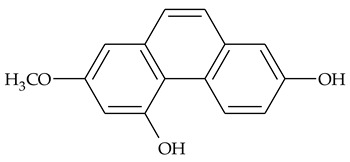 4,7-dihydroxy-2-methoxyphenanthrene **(16)**	R_f_, ^1^H-NMR, ^13^C-NMR, MS	[9]
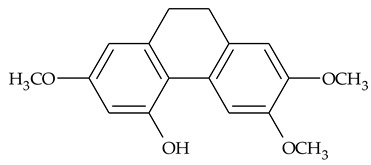 4-hydroxy-2,6,7-trimethoxy-9,10-dihydrophenanthrene **(17)**	m.p., UV, MS, ^1^H-NMR, ^13^C-NMR	[23]
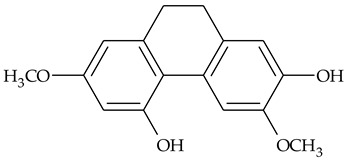 4,7-dihydroxy-2,6-dimethoxy-9,10-dihydrophenanthrene **(18)**	m.p., UV, MS, ^1^H-NMR, ^13^C-NMR	[23]
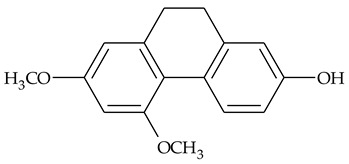 Orchinol **(19)**	Rf, UV, MS, ^1^H-NMR, ^13^C-NMR	[9]
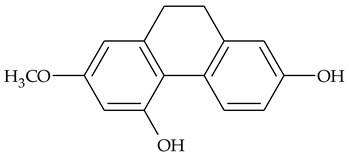 Lusianthridin **(20)**	Rf, UV, MS, ^1^H-NMR, ^13^C-NMR	[9]
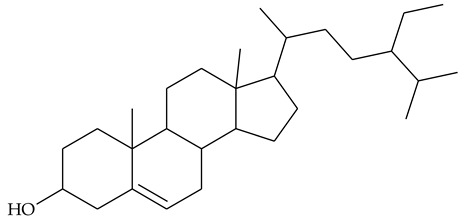 β-sitosterol **(21)**	m.p., 1H-NMR, MS	[24]
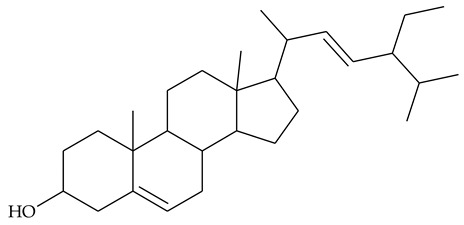 Stigmasterol **(22)**	m.p., 1H-NMR, MS	[24]
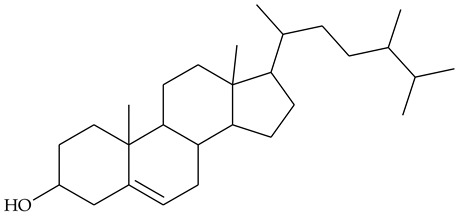 Campesterol **(23)**	m.p., 1H-NMR, MS	[24]
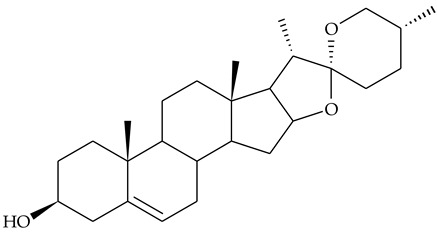 Diosgenin **(24)**	HPLC	[3]
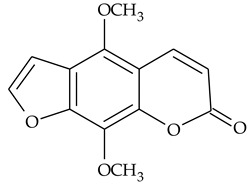 5,8-dimethoxypsoralen **(25)**	MS,^1^H-NMR, ^13^C-NMR	[16]
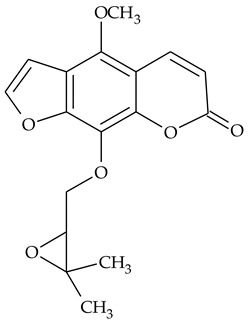 Heraclinin **(26)**	MS,^1^H-NMR, ^13^C-NMR	[16]

m.p.—melting point, R_f_—retention factor.

**Table 2:** Concentrations of some phenolic compounds of TCR extract in CHCl_3_.

Compound	Concentration (in mg Per 1 g Dry Extract)
Total phenols	167 GAE
Phenolic acids	
Gallic acid	0.43
Protocatehuic acid	BLD
Chlorogenic acid	ND
Vanillic acid	0.89
Caffeic acid	NF
Syringic acid	2.95
p-Coumaric acid	BLD
Ferulic acid	0.21
Salicylic acid	0.27
Rosmarinic acid	0.41
Flavonoids	15.6 QE
Rutin	ND
Hesperidin	ND
(+)-Catechin	0.54
(−)-Epicatechin	0.72
Quercetin	0.38
Kaempherol	9.98
Tannins	12.64 TAE

GAE, gallic acid equivalents; QE, quercetin equivalents, TAE, tannic acid equivalents; ND, not detected; BLD, below the limit of detection.

**Table 3:** Biological activity of *Tamus communis* rhizome extracts and some of individual phytochemicals.

Extract/Compound	Model Used	Biological Activity	Reference
Extract in 80% EtOH	Rats, oral administration	Anti-inflammatory, analgesic	[13]
Extract in 80% EtOH	Rats (cotton pellet-induced granuloma)	Anti-inflammatory	[15]
1% extract in EtOH	Rats (carrageenan-induced edema)	Anti-inflammatory	[42]
Extract in EtOH and H_2_O	Rats, oral administration	Anti-inflammatory, antinocieptive	[3]
**7, 13, 14**	HeLa cell line *	Cytotoxicity	[8]
Extract in 80% EtOH	Hep-2 & AMN-3 cell lines *	Cytotoxicity	[3]
**24**	Hep-2 & AMN-3 cell lines *	Cytotoxicity	[3]
Extract in CHCl_3_	*S. aureus, Bacillus sp, E. agglomerans, S. marcescens*	Antibacterial	[7]
Extract in AcOEt	*S. aureus, E. agglomerans, S. marcescens*	Antibacterial	[7]
**1, 2, 3, 4, 17, 18**	Cells infected with VSV or HRV 1B *	Antiviral	[46]
Extracts in MeOH, CHCl_3_ or AcOEt	Xanthine oxidase	Enzyme inhibition	[31]
**25, 26**	Xanthine oxidase	Enzyme inhibition	[16]
**11**	Acetylcholine esterase	Enzyme inhibition	[9]
Extract in Et_2_O	Butyrylcholine esterase	Enzyme inhibition	[9]
**14**	Butyrylcholine esterase	Enzyme inhibition	[9]
Extract in 85% MeOH	Mice, intraperitoneal administration	Improvement in antioxidant status	[12]

* HeLa, cervix adenocarcinoma cells; HEp-2, human epidermoid laryngocarcinoma cells; AMN-3, mammary adenocarcinoma cells; VSV, vesicular stomatitis virus; HRV 1B, human rhinovirus serotype.

## Data Availability

Not applicable.
